# 两种扩增体系NK细胞生物学特性比较及治疗异基因造血干细胞移植后白血病复发的初步研究

**DOI:** 10.3760/cma.j.issn.0253-2727.2022.05.009

**Published:** 2022-05

**Authors:** 勋红 曹, 志东 王, 于谦 孙, 军 孔, 晟晔 卢, 菲菲 唐, 圆圆 张, 景枝 王, 兰平 许, 晓辉 张, 昱 王, 开彦 刘, 晓军 黄, 翔宇 赵

**Affiliations:** 北京大学人民医院，北京大学血液病研究所，国家血液系统疾病临床医学研究中心，北京 100044 Peking University People's Hospital & Peking University Institute of Hematology, National Clinical Research Center for Hematologic Disease, Beijing 100044, China

**Keywords:** 白血病, 复发, 扩增, NK细胞, Leukemia, Relapse, Expansion, NK cells

## Abstract

**目的:**

比较不同扩增体系NK细胞生物学特性的差异以及治疗异基因造血干细胞移植后（allo-HSCT）白血病复发的疗效。

**方法:**

分别采用CD3/CD52单抗扩增法和饲养层细胞扩增法诱导供者来源NK细胞大量扩增，检测扩增前后NK细胞表型、因子分泌、细胞毒的变化规律；选取16例allo-HSCT后复发白血病患者，8例输注CD3/CD52单抗扩增NK细胞，8例输注饲养层细胞扩增NK细胞，观察患者的治疗反应和长期生存情况。

**结果:**

①与CD3/CD52单抗扩增体系相比，饲养层细胞扩增体系NK细胞纯度较高、NK细胞表面活化性受体DNAM-1及NKp30表达较高、抑制性受体CTLA-4表达较低，而两种NK细胞NKG2D/CD25/CD69/Trail/PD-1/TIM-3/TIGIT表达差异无统计学意义。②两种扩增体系NK细胞Ki-67指数均明显增加，以饲养层细胞扩增NK细胞尤为明显；饲养层细胞扩增NK细胞穿孔素及颗粒酶B表达水平均明显高于CD3/CD52单抗扩增NK细胞。③16例allo-HSCT后复发白血病患者NK细胞输注过程中均未观察到明显不良反应。NK细胞输注后中位随访时间为2554（917～2583）d，CD3/CD52单抗扩增组中3例患者无白血病存活，5例死亡；饲养层细胞扩增组中6例患者长期存活，2例死亡。5例患者在NK细胞输注前存在移植物抗宿主病，NK细胞输注后移植物抗宿主病未加重甚至缓解。

**结论:**

CD3/CD52单抗扩增与饲养层细胞扩增体系NK细胞的生物学特性具有明显差异；NK细胞输注对allo-HSCT后复发白血病患者的疗效仍需要进一步验证。

## Abstract (en)

**Objective:**

To explore the differences in the biological effects of different expansion systems on natural killer（NK）cells, as well as the safety and preliminary clinical efficacy in the treatment of patients with recurrence after allogeneic hematopoietic stem cell transplantation（allo-HSCT）.

**Methods:**

Peripheral blood cells from healthy donors were stimulated with either CD3 combined with CD52 or K562 feeder cells loaded with IL-21/4-1BB to induce NK cell expansion. Changes in the NK cell phenotype, cytokine secretion, and cytotoxicity before and after expansion were detected. We also evaluated the safety and clinical efficacy of two different expansion strategies for patients received NK infusion.

**Results:**

Compared with the CD3/CD52 monoclonal antibody amplification system, the feeder cell expansion group had a higher purity of NK cells and higher expression ratios of NK cell surface activation receptors such as DNAM-1 and NKp30, while inhibitory receptor CTLA-4 expression was low and NKG2D/CD25/CD69/ Trail/PD-1/TIM-3/TIGIT had no statistically significant differences between the groups. Further functional results showed that the expression level of KI67 in NK cells after expansion in the two groups increased significantly, especially in the feeder cell expansion group. Simultaneously, the perforin and granzyme B levels of NK cells in the feeder cell expansion group were significantly higher than in the CD3/CD52 expansion group. A retrospective analysis of eight patients who received monoclonal antibody-expanded NK cell reinfusion and nine patients with trophoblast cell-expanded NK cell reinfusion was done. The disease characteristics of the two groups were comparable, NK cell reinfusion was safe, and there were no obvious adverse reactions. Clinical prognostic results showed that in the CD3/CD52 monoclonal antibody amplification group, the MRD conversion rate was 50％（2/4）, and the feeder cell expansion group was 50％（3/6）. After 5 years of follow-up from allo-HSCT, three patients in the monoclonal antibody expansion group had long-term survival without leukemia, and the remaining five patients had died; two patients died in the feeder cell expansion group, and the other six patients had long-term survival. Six cases had GVHD before NK cell reinfusion, and GVHD did not aggravate or even relieved after NK cell reinfusion.

**Conclusion:**

Preliminary results show that the biological characteristics of NK cells with diverse expansion strategies are significantly different, which may affect the clinical prognosis of patients with recurrence or persistent minimal residual disease after HSCT. The two groups of patients treated with NK cells from different expansion strategies had no obvious adverse reactions after NK cell infusion, but efficacy still needs to be further confirmed.

异基因造血干细胞移植（allo-HSCT）是白血病的有效治疗手段，移植后复发是影响移植疗效的主要原因。目前，移植后白血病复发的常规治疗方法包括化疗及供者淋巴细胞输注（DLI），但DLI可使移植物抗宿主病（GVHD）的发生风险增加[1]。因此，探讨新的复发后治疗方法是提高白血病患者移植疗效的关键。

自然杀伤（NK）细胞是构成机体重要的天然免疫屏障之一，无需致敏即可杀伤肿瘤细胞或感染的细胞，通过分泌穿孔素、颗粒酶等介导靶细胞杀伤[2]–[3]，另一方面可通过分泌IFN-γ、TNF-α等活化适应性免疫系统而发挥协同免疫防御作用[4]。除此之外，抗体依赖的细胞介导细胞毒性作用（ADCC）也是NK细胞效应功能发挥的重要途径。

NK细胞效应功能强度受NK细胞活化性受体与抑制性受体的平衡、NK细胞的成熟度及对细胞因子的响应体内外多种因素调控。内源性NK细胞往往受疾病及自身HLA分子抑制等多方面影响，数量及效应功能受损，但研究证明来自健康供者的NK细胞具有杀伤肿瘤细胞功能[5]。然而，若输注的NK细胞数量少、疗程短，患者仍面临肿瘤复发的风险。目前，CD3/CD52单抗法和K562饲养层细胞法是临床应用较多的两种NK细胞体外扩增方法[6]–[7]。本研究比较了以上两种扩增体系NK细胞的生物学特性，并回顾性分析比较了两种扩增体系NK细胞过继性输注治疗allo-HSCT后白血病复发患者的安全性及初步疗效。

## 对象与方法

一、NK细胞扩增方法

采集健康供者外周血，提取单个核细胞，CD3/CD52单抗扩增法参照文献[6]，饲养层细胞扩增法参照文献[8]。

二、NK细胞采集时间点

NK细胞扩增前采外周血检测表型及功能，体外扩增2周后检测扩增后NK细胞表型及功能各项指标。

三、NK细胞表型及功能评估方法

1. 表型评估：外周血来源的单个核细胞及扩增后的NK细胞，加入膜表面抗体标记，避光孵育30 min，溶血并PBS洗涤后上机检测。抗体选择见表1。

2. 功能评估：对于NK细胞扩增前后细胞增殖及细胞毒的评估，则在膜表面抗体标记后，按照Cytofix/Cytoperm试剂盒（美国Becton Dickinson公司产品）说明固定破膜后标记细胞内免疫因子。抗体选择见表1。

**表1:** 流式细胞术评估NK细胞表型及功能所用抗体

生产商	抗体	标志抗体	克隆号
BD	CD56	BUV737	NCAM16.2
BD	CD158a	BV421	HP-3E4
BD	CD45	V500	HI30
Biolegend	NKG2D	BV605	1D11
Biolegend	CXCR3	BV650	G025H7
Biolegend	CTLA-4	BV785	BNL3
BD	CD158e	FITC	DX9
Biolegend	CD158f	PE	UP-R1
Biolegend	CD57	PE-CF594	12G5
BD	PD-1	PE-Cy7	EH12.1
MACS	NKG2C	AF647	FAB138N
Biolegend	CD3	AF700	UCHT1
BD	CD16	APC-Cy7	3G8
BD	CD25	BUV395	2A3
BD	CD69	BUV737	FN50
Biolegend	4-1BB	BV421	4B4-1
Biolegend	CXCR4	BV605	12G5
Biolegend	CD27	BV650	O323
BD	TIM-3	BV711	7D3
Biolegend	LFA-1	FITC/AF488	m24
BD	CD94	Percp-Cy5.5	HP-3D9
BD	NKP46	PE	9E2/NKp46
Biolegend	CD96	PE-CF594	NK92.39
BD	CD62L	PE-Cy7	DREG-56
BD	DNAM-1	APC/AF647	DX11
Biolegend	CD56	APC-Cy7	HCD56
BD	NKP30	BV421	p30-15
Biolegend	TIGIT	BV605	A15153G
Biolegend	CX3CR1	BV650	2A9-1
BD	CD107a	BV786	H4A3
RD	TRAIL	FITC	FAB687G
Biolegend	NKP44	Percp-Cy5.5	P44-8
MACS	NKG2A	PE-Cy7	REA110
Biolegend	NKp80	APC/AF647	5D12

四、入组病例及移植方案

1. 病例：16例患者纳入本研究。8例患者输注CD3/CD52扩增NK细胞，均为2011年2月至2014年2月在北京大学血液病研究所进行allo-HSCT且移植后白血病复发患者；8例患者输注K562饲养层扩增NK细胞，为2016年8月至2018年6月在北京大学血液病研究所进行allo-HSCT且移植后白血病复发患者。所有患者均签署知情同意书。

2. 预处理方案：全部采用常规的改良BU/CY（白消安/环磷酰胺）方案，HLA配型不合患者移植预处理方案中加用抗胸腺细胞球蛋白（ATG）[9]。

3. 干细胞的动员和采集：全部患者采用骨髓联合外周血干细胞移植，所有患者均以G-CSF动员5～6 d（−3 d开始），剂量为5 µg/kg[10]–[11]。

4. 急性GVHD的预防：采用环孢素A（CsA）联合霉酚酸酯（MMF）及短程甲氨蝶呤（MTX）方案。

5. 植活标准：连续3 d中性粒细胞绝对计数（ANC）≥0.5×10^9^/L为粒细胞植活，PLT≥20×10^9^/L连续7 d且脱离血小板输注为血小板植活[12]。

五、患者移植后微小残留病（MRD）监测

多参数流式细胞术（MFC）和实时荧光定量PCR（RT-PCR）用来检测MRD[13]。MRD阳性：①MFC连续两次阳性或者WT1连续两次阳性抑或是在同一标本中MFC或WT1阳性各1次；②移植后出现基因重排或突变。MRD缓解：在NK细胞输注后达到MRD阴性状态并维持至少1个月[14]。对于骨髓中原始细胞>5％定义为血液学复发。

六、随访

通过查阅住院/门诊病历及电话随访方式获取患者生存资料。

七、统计学处理

采用GraphPad Prism8分析数据，采用非配对*t*检验进行单因素分析，*P*<0.05为差异有统计学意义。

## 结果

一、两种扩增体系NK细胞生物学差异

1. 两种扩增体系NK细胞活化性受体的表达：CD3/CD52单抗扩增组NK细胞在CD45阳性淋巴亚群中的中位占比为18.9％，而饲养层细胞扩增的NK细胞中位数比例为85.0％，纯度更高。通过对不同体系扩增前后NK细胞活化性受体的表达评估，我们发现滋养层扩增的NK细胞的活化性受体DNAM-1及NKp30的表达都明显高于CD3/CD52单抗扩增组（*P*＝0.016，*P*＝0.006），而NKG2D、Trail占NK细胞的百分比在两组间差异无统计学意义（*P*>0.05）（图1、图2）。CD25及CD69分别被认为是NK细胞早期活化和晚期活化的重要指标[15]。在我们的队列中，CD25及CD69占NK细胞百分比在CD3/CD52单抗扩增组及饲养层扩增组均无明显差别（*P*>0.05），提示两种扩增NK细胞尽管在活化性受体表达方面存在差异，但两者综合的活化程度相当（图1、图2）。

**图1:**
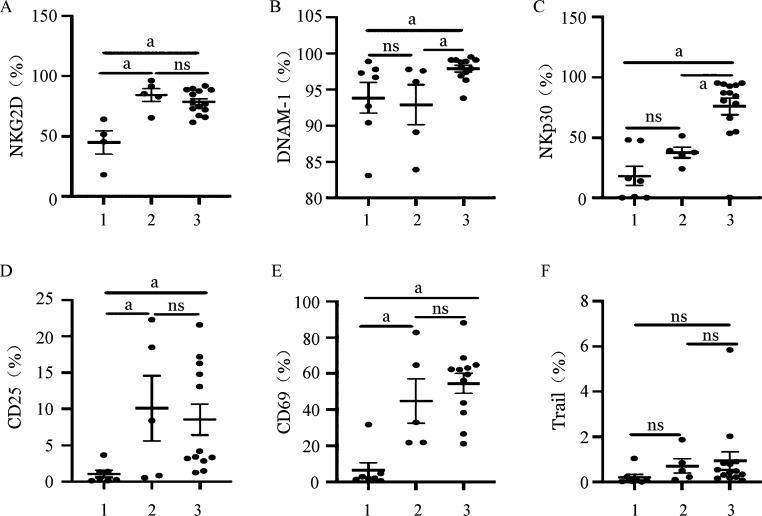
两种不同扩增体系NK细胞活化性受体表达 1、2、3分别为扩增前组、CD3/CD52单抗扩增组、饲养层细胞扩增组。ns：差异无统计学意义；^a^ *P*<0.05

**图2:**
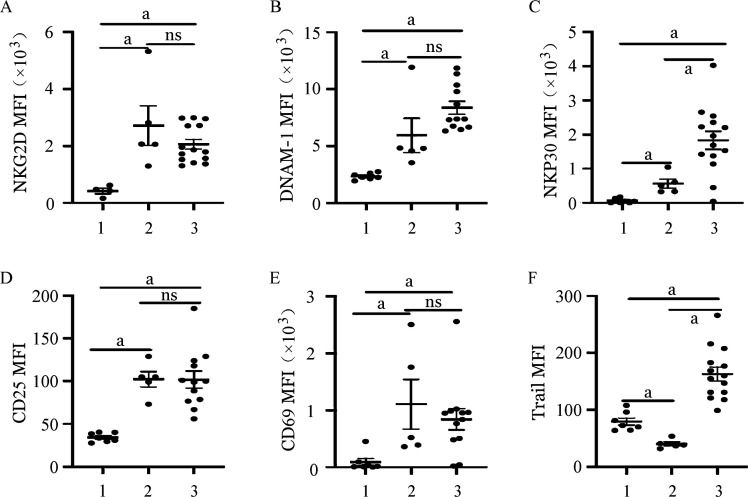
两种不同扩增体系NK细胞活化性受体平均荧光强度（MFI） 1、2、3分别为扩增前组、CD3/CD52单抗扩增组、饲养层细胞扩增组。ns：差异无统计学意义；^a^ *P*<0.05

2. 两种扩增体系NK细胞抑制性受体的表达：两种体系扩增的NK细胞抑制性受体CTLA4、PD-1、TIM-3的平均荧光强度（MFI）较扩增前均明显上调（*P*<0.001，*P*＝0.003，*P*＝0.001），然而Tigit的平均荧光强度无明显变化（*P*>0.05）（图3）。饲养层细胞扩增组NK细胞CTLA4（占NK细胞百分比）的表达明显低于CD3/CD52单抗扩增组（*P*<0.001），而其他抑制性受体（PD-1、TIM-3、Tigit）在两组间无论是平均荧光强度还是在NK细胞中占比差异均无统计学意义（*P*>0.05）。提示体外扩增诱导NK细胞活化的同时可能一定程度上伴随功能耗竭。

**图3:**
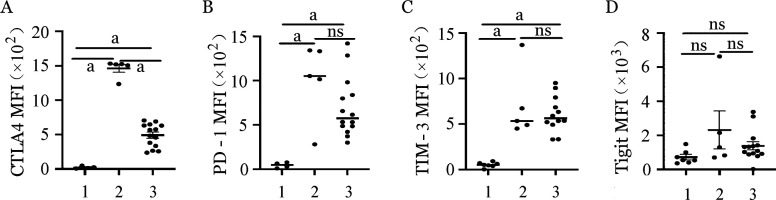
两种不同扩增体系NK细胞抑制性受体平均荧光强度（MFI） 1、2、3分别为扩增前组、CD3/CD52单抗扩增组、饲养层细胞扩增组。ns：差异无统计学意义；^a^ *P*<0.05

3. 两种扩增体系NK细胞趋化型受体的表达：NK细胞向组织脏器中的迁移依赖于其表面的各种黏附性分子及趋化性受体的表达。为此，我们评估了不同扩增体系对NK细胞表面黏附性分子及趋化性受体表达的影响，结果显示在饲养层细胞扩增组NK细胞中CXCR3、CD96的比例明显高于CD3/CD52单抗扩增组（*P*＝0.004，*P*＝0.037），CX3CR1及4-1BB在NK细胞的表达比例在饲养层扩增组更低，CXCR4、CD94、CD27及CD62L的比例在两种扩增体系NK细胞间差异均无统计学意义（*P*>0.05），提示从黏附分子和趋化受体表达角度，两种扩增方法各有利弊（图4）。

**图4:**
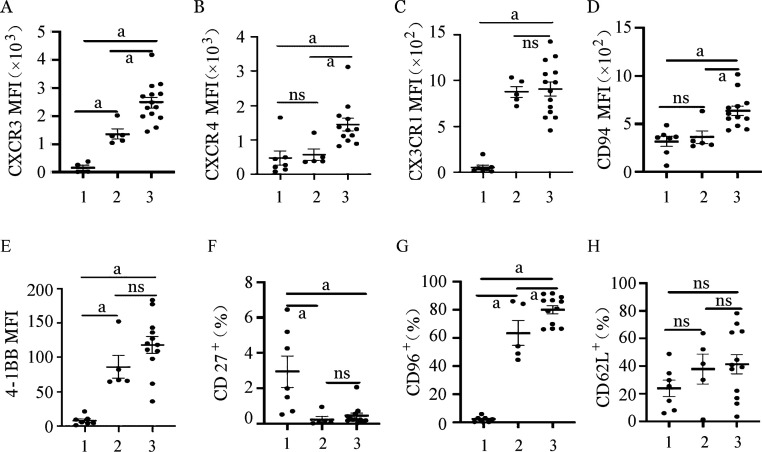
两种不同扩增体系NK细胞趋化性受体表达 1、2、3分别为扩增前组、CD3/CD52单抗扩增组、饲养层细胞扩增组。MFI：平均荧光强度；ns：差异无统计学意义；^a^ *P*<0.05

4. 两种扩增体系NK细胞功能表达差异：我们通过检测扩增NK细胞的Ki-67指标评估其不同体系的扩增能力。结果显示，饲养层扩增NK细胞的增殖潜能明显高于CD3/CD52单抗扩增NK细胞（*P*＝0.033），穿孔素及颗粒酶B的表达水平也明显高于抗体扩增NK细胞（图5）。

**图5:**
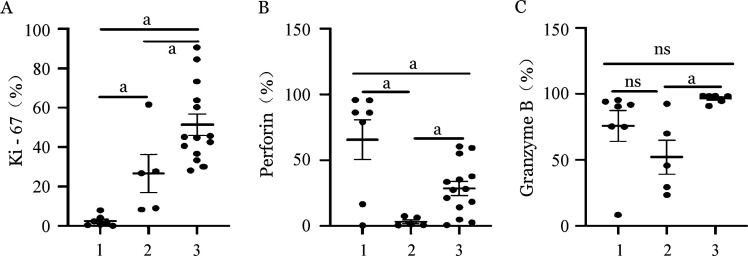
两种不同扩增体系NK细胞生物学功能比较 1、2、3分别为扩增前组、CD3/CD52单抗扩增组、饲养层细胞扩增组。MFI：平均荧光强度；ns：差异无统计学意义；^a^ *P*<0.05。Perforin：穿孔素；Granzyme B：颗粒酶B

二、入组患者的临床特点

CD3/CD52单抗扩增组中男5例、女3例，4例接受同胞HLA全相合allo-HSCT，4例接受haplo-HSCT，7例患者移植时处于完全缓解状态，1例移植前为复发状态。至2021年6月随访终止时，中位随访时间为2 554 d，共有3例患者死亡。

饲养层扩增组中男4例、女4例，5例接受同胞HLA全相合allo-HSCT，3例接受haplo-HSCT，8例患者移植时均处于完全缓解状态。患者首次接受供者来源NK细胞输注的中位时间是移植后482 d。至2021年6月随访终止时，中位随访时间1 015.5（917～1 068）d，共有2例患者死亡。

患者主要临床特点和NK细胞治疗前情况见表2。

**表2:** 16例异基因造血干细胞移植后复发白血病患者的一般资料及NK细胞治疗前情况

例号	性别	年龄	疾病类型	移植模式	急性GVHD	NK细胞输注前化疗方案	NK细胞输注前疾病状态
1	男	37	AML-M2	HLA全相合	无	FLAG，HAA	血液学复发
2	男	52	B-ALL	单倍型	无	COPD	血液学复发
3	男	26	AML-M2	HLA全相合	无	HAA	髓外复发
4	女	12	AML-M2	单倍型	Ⅰ度，皮肤Ⅱ度	无	MRD（+）
5	男	21	MDS-RAEB2	单倍型	Ⅳ度（皮肤、肝）	无	MRD（+）
6	女	46	AML-M2	单倍型	无	无	MRD（+）
7	男	47	B-ALL	HLA全相合	无	COPD	髓外复发，MRD（+）
8	女	33	AML-M2	HLA全相合	肠道Ⅰ度	无	血液学复发
9	女	40	AML-M2	HLA全相合	无	无	MRD（+）
10	男	31	AML-M2	单倍型	Ⅰ度，皮肤Ⅱ度	AA	MRD（+）
11	女	54	AML-M4	HLA全相合	无	无	血液学复发，髓外复发，MRD（+）
12	女	24	AML-M5	单倍型	皮肤Ⅰ度	AA	MRD（+）
13	男	31	AML-M2	单倍型	无	无	血液学复发
14	男	30	AML-M2	HLA全相合	无	FLAG	MRD（+）
15	男	49	AML-M2	HLA全相合	无	无	血液学复发
16	女	33	AML-M2	HLA全相合	无	无	MRD（+）

注：AML：急性髓系白血病；B-ALL：急性B淋巴细胞白血病；MDS-RAEB2：骨髓增生异常综合征-难治性贫血伴有原始细胞增多2型；MRD：微小残留病；FLAG方案：氟达拉宾+阿糖胞苷+G-CSF；HAA方案：高三尖杉酯碱+阿糖胞苷+阿柔比星；COPD方案：环磷酰胺+长春新碱+柔红霉素+泼尼松；AA方案：阿柔比星+阿糖胞苷；GVHD：移植物抗宿主病

三、安全性评价

两组患者在NK细胞输注后均未观察到发热、血象下降、脏器功能损害等不良反应。

四、NK细胞治疗情况及疗效评价

饲养层细胞扩增组平均接受NK细胞输注次数为1.25次，CD3/CD52单抗扩增组为2.4次。两组共观察到血液学复发5例（伴髓外复发1例），单纯MRD持续阳性8例，单纯髓外复发1例，髓外复发合并MRD阳性1例。患者NK细胞中位输注次数为1次，移植后接受NK细胞输注治疗的中位时间为移植后434（103～1 225）d。NK细胞输注后中位随访时间为2 554（917～2 583）d，复发患者缓解率为16.7％（1/6），MRD阳性患者转阴率为54.5％（6/11），无白血病生存（LFS）率为43.75％（7/16），总生存（OS）率为56.25％（9/16）。对于持续缓解的患者，NK细胞治疗后有6例患者接受化疗联合DLI，4例行2次移植。

饲养层扩增组8例患者中6例有治疗反应，其中HLA全相合移植4例，haplo-HSCT 2例，输注过程均无明显不良反应，其中5例患者至今无白血病长期生存，1例患者因血液复发死亡。CD3/CD52单抗扩增组8例患者中4例有治疗反应，其中HLA全相合移植2例，haplo-HSCT 2例，输注过程均无明显不良反应，其中3例患者至今无白血病长期生存，1例因重症肺炎导致呼吸衰竭而死亡。

而对NK细胞回输治疗无反应患者中，CD3/CD52单抗扩增组8例患者中4例无治疗反应（1例为持续MRD阳性，3例为血液学复发），经化疗、DLI等治疗均未得到缓解，4例均死亡（死于中枢神经系统感染、重度GVHD合并TMA各1例，血液学复发2例）。在饲养层细胞扩增组8例患者中有2例无治疗反应（均为MRD持续阳性），经化疗后分子学及血液学复发指标均未得到缓解，其中1例NK细胞输注后MRD仍持续阳性，但后续再行DLI治疗后MRD转阴，至今无白血病生存；另一例患者死于髓外复发。

CD3/CD52单抗扩增组、饲养层扩增组NK细胞输注后MRD转阴率分别为50％（2/4）、50％（3/6）。

滋养层扩增组的例11在接受NK细胞治疗前有重度慢性GVHD（累及肺、皮肤、口腔等器官）；另有3例在移植后分别表现为皮肤、口腔及指甲、皮肤及肺部急性GVHD表现，输注后症状缓解。

在CD3/CD52单抗扩增组，我们观察到3例患者GVHD症状在NK细胞输注后缓解，提示NK细胞可能有抗GVHD的功能。NK细胞治疗情况及疗效见表3。

**表3:** 16例异基因造血干细胞移植后复发白血病患者的NK细胞治疗情况及随访结果

例号	NK细胞输注次数	NK细胞输注量（×10^9^/L）	疗效评估	随访时间（d）	随访结果
1	2	6.20	无效	2 560	死亡（中枢神经系统感染）
2	1	4.76	无效	2 560	死亡（重度GVHD/TMA）
3	5	6.62	有效	2 583	存活
4	1	4.19	有效	2 540	存活
5	3	6.02	有效	2 540	存活
6	1	0.36	无效	2 554	死亡（血液学复发）
7	5	10.31	有效	2 554	死亡（重症肺炎）
8	1	6.44	无效	2 552	死亡（血液学复发）
9	1	6.18	有效	1 068	存活
10	2	7.02	有效	1 064	存活
11	1	7.10	有效	1 019	存活
12	1	4.40	无效	917	存活
13	1	7.32	有效	1 012	死亡（血液学复发）
14	2	3.14	无效	994	死亡（血液学复发）
15	1	3.82	有效	917	存活
16	1	2.48	有效	1 047	存活

注：GVHD：移植物抗宿主病；TMA：血栓性微血管病

## 讨论

NK细胞是机体天然免疫屏障的重要组成，是抗病毒和抗肿瘤的主力军之一[7]。除此之外，供者KIR不相合的NK细胞输注至受者体内，可通过抑制受者来源的抗原提呈细胞及T细胞，降低移植物抗宿主病的发生。因此，过继性输注NK细胞的免疫疗法已成为了潜在抗肿瘤的有效治疗方式。然而，由于外周血NK细胞比例低，获取数量少，使得NK细胞治疗疗效受限。体外细胞扩增体系的发展使得NK细胞可在短期（2～3周）内获取，并且输注的NK细胞在动物及人体中均显示发挥抗肿瘤效应的同时不增加GVHD的发生率[5]。但是，不同扩增体系产出的NK细胞生物学特性和临床疗效存在较大差异[16]–[17]。Masuyama等[6]首次通过CD3/CD52单抗体外刺激外周血单个核细胞后联合自体血浆及IL-2，在无饲养层细胞体系中实现了NK细胞的量级扩增，且抗肿瘤功能增强。尽管该无饲养层体系扩增NK细胞比例相对较高，但培养14 d后的扩增细胞中CD8^+^T细胞及CD4^+^T细胞比例中位值分别为60％、15％。随着体外扩增体系的完善，体外饲养层细胞扩增体系扩增NK细胞效率更佳[14]。然而体外饲养层细胞扩增体系需要大量且持续的因子刺激，当输注至体内后，细胞因子刺激骤失将导致输注后NK细胞代谢加快。本研究首次对CD3/CD52单抗扩增NK细胞与K562饲养层扩增NK细胞的生物学差异进行探讨，结果显示，以K562饲养层扩增的NK细胞纯度较高、T细胞含量较低。两种不同扩增体系NK细胞活化性受体表达及KI-67均明显高于扩增前NK细胞，说明NK细胞扩增同时伴随着功能活化，尤以饲养层扩增组NK细胞的增殖水平及活化性受体DNAM-1及NKP30的表达水平更高，而抑制性受体CTLA4表达明显低于CD3/CD52单抗扩增组，抑制性受体PD-1、Tigit及Tim-3的表达在两组间无明显差异。以上结果提示饲养层扩增的NK细胞的抗肿瘤能力可能更强。值得注意的是，两组扩增后NK细胞的活化性受体明显增加，抑制性受体相较于扩增前组也明显上调，这与Judge等[18]的报道一致，可能是扩增NK细胞为了防止过度活化的适应性调控。

既往有研究前瞻性评估不同体系扩增的NK细胞在治疗AML中的疗效，结果提示过继性NK细胞输注在治疗低白血病负荷中有优势[8],[14]，然而尚未有研究报道不同体系扩增的NK细胞在治疗AML中的疗效差异。在本研究中，两种不同扩增体系NK细胞在生物学特性上存在差异，两种扩增体系NK细胞输注对移植后血液学复发白血病患者均无显著疗效；而对持续MRD阳性患者来说，在CD3/CD52单抗扩增组和饲养层扩增组，分别有50％（2/4）、50％（3/6）的MRD阳性患者NK细胞治疗后转阴，提示NK细胞治疗可能有清除体内MRD作用，并且以饲养层扩增NK细胞效果更好，这可能与饲养层扩增的NK细胞活化性受体表达更高有关；与此同时，本研究结果表明饲养层扩增NK细胞趋化型因子受体CXCR3与CXCR4的表达均明显高于CD3/CD52单抗扩增NK细胞，提示饲养层扩增NK细胞的迁移速度与向骨髓的趋化能力更高，也更有利于扩增NK细胞向肿瘤部位的迁移[19]。同时，Denman等[20]的研究表明装载IL-21的K562细胞作为饲养层细胞体外扩增NK细胞的端粒酶更长，提示滋养层扩增NK细胞可能体内存活时间更长。

在本研究中，两种扩增体系NK细胞治疗移植后复发白血病患者并未引起或加重GVHD，且两组均有部分患者的急慢性GVHD症状在NK细胞输注后得以缓解，提示NK细胞输注治疗的安全性良好。既往研究表明NK细胞在抗白血病细胞的同时具有抗GVHD效应[5]，这得益于NK细胞对效应性T细胞的功能和数量制约：一方面NK细胞可杀伤供者来源的T细胞，同时可通过杀伤不成熟的树突状细胞减少抗原提呈[21]–[22]。与此同时，异基因来源的NK细胞不损伤患者的靶器官，因此不会发生GVHD和细胞因子风暴（CRS）。

本研究结果初步显示，CD3/CD52单抗扩增与饲养层细胞扩增体系NK细胞的表型和功能具有明显差异，两种扩增体系NK细胞在治疗移植后持续MRD阳性均有效。本研究为回顾性研究且队列样本数少，因此NK细胞输注对allo-HSCT后复发白血病患者的疗效仍需要进一步验证。
